# Potential Application of Luteolin as an Active Antibacterial Composition in the Development of Hand Sanitizer Products

**DOI:** 10.3390/molecules27217342

**Published:** 2022-10-28

**Authors:** Meihua Xi, Yujie Hou, Ruolin Wang, Minhui Ji, Yingying Cai, Jingfang Ao, Heyu Shen, Mei Li, Jun Wang, Anwei Luo

**Affiliations:** College of Food Science and Engineering, Northwest A&F University, Xianyang 712100, China

**Keywords:** luteolin, *E. coli*, *S. aureus*, antibacterial activity, antibacterial hand sanitizer

## Abstract

Antibacterial hand sanitizers could play a prominent role in slowing down the spread and infection of hand bacterial pathogens; luteolin (LUT) is potentially useful as an antibacterial component. Therefore, this study elucidated the antibacterial mechanism of LUT against *Escherichia coli* (*E. coli*) and *Staphylococcus aureus* (*S. aureus*) and developed an antibacterial hand sanitizer. The results showed that LUT had excellent antibacterial activity against both *E. coli* (minimum inhibitory concentration (MIC) = 312.5 μg/mL, minimal bactericidal concentration (MBC) = 625 μg/mL), and *S. aureus* (MIC = 312.5 μg/mL, MBC = 625 μg/mL). Furthermore, LUT induced cell dysfunction in *E. coli* and *S. aureus*, changed membrane permeability, and promoted the leakage of cellular contents. Confocal laser scanning microscopy (CLSM) and scanning electron microscopy (SEM) analysis showed that LUT treatment affected cell structure and disrupted cell membrane integrity. The Fourier transform infrared analysis (FTIR) also confirmed that the LUT acted on the cell membranes of both *E. coli* and *S. aureus*. Overall, the application of LUT in hand sanitizer had better inhibition effects. Therefore, this study could provide insight into expanding the application of LUT in the hand sanitizer markets.

## 1. Introduction

Hands, as one of the main ways in which we have contact with the world, come in contact with various microorganisms presented outside. Hands are usually exposed to bacteria, including *Escherichia coli* (*E. coli*), *Staphylococcus aureus* (*S. aureus*), and Gram-negative and Gram-positive bacteria, respectively, which generally cause gastrointestinal diseases and infections in humans, promoting severe public health consequences [1]. Therefore, hand hygiene is essential to reducing microbial burdens, transmission, and infections. Efforts are now directed toward alleviating the current costs and seeking a green transformation through the development of new raw materials, by using natural plants and their by-products as the main components, or by using readily biodegradable and pollution-free materials to produce daily chemical products.

Many secondary metabolites produced by normal metabolic pathways in plants have potential antimicrobial activities. Compounds, including polyphenols, flavonoids, quinones, and terpenoids, can target bacteria by changing membrane permeability, inhibiting the synthesis of enzymes, or blocking biochemical reactions [2,3,4]. Among these compounds, it has been described that flavonoids can exert their antibacterial effects, namely by direct killing of bacteria, synergistic activation of antibiotics, and reducing bacterial pathogenicity [5]. In addition, flavonoids can inactivate efflux pumps, disrupt the cytoplasmic membrane, and inhibit β-lactamase and topoisomerase, preventing the development of bacterial resistance to antibiotics [6]. Therefore, flavonoids in food or daily chemical products can improve consumer acceptance, and enhance the utilization of plants and their by-products.

Luteolin (LUT) is a metabolite belonging to flavonoids, which exists widely in many plants and their by-products, and is one of the most bioactive flavonoids [7]. LUT has received much attention for its excellent anti-cancer and anti-inflammatory activities [8,9]. LUT could be used to treat various cancers, such as lung and breast cancers [10]. This compound could hamper the progression of cancer through multiple mechanisms, including the suppression of kinases, regulation of the cell cycle, induction of apoptotic cell death, and reduction of transcription factors. The anti-inflammatory effects of LUT are through the inhibition of signal transduction pathways by regulating inflammatory mediators and different cytokines [11]. In addition, LUT has been shown to have effective antimicrobial activity against foodborne pathogens, such as *S. aureus*, *E. coli*, and *Salmonella* [12]. Qian and collaborators [13] investigated the antibacterial and biofilm activities of LUT against *S. aureus* and *Listeria monocytogenes* and found that LUT could damage the cell membranes of these two bacteria and inhibit biofilm formation. The authors suggested that LUT may have valuable applications in the food industry as a food preservative and surface disinfectant. Furthermore, LUT has already been commercially developed as a health food and can be found in cosmetic products, and non-toxic side effects were detected in mice and rats (the oral median lethal dose (LD_50_) was greater than 2500 and 5000 mg/kg, respectively, corresponding to 219.8–793.7 mg/kg in humans, approximately) [14]. Although several studies have presented viable theoretical support, reports linking the antibacterial activity of LUT to products with practical life applications are still insufficient.

Therefore, this study evaluated the antibacterial activity and mechanism of action of LUT against *E. coli* and *S. aureus* and developed an antibacterial hand sanitizer product. This study could provide new insight into the development of antibacterial daily chemical products in the future.

## 2. Results and Discussion

### 2.1. Antibacterial Activity

**Figure 1:**
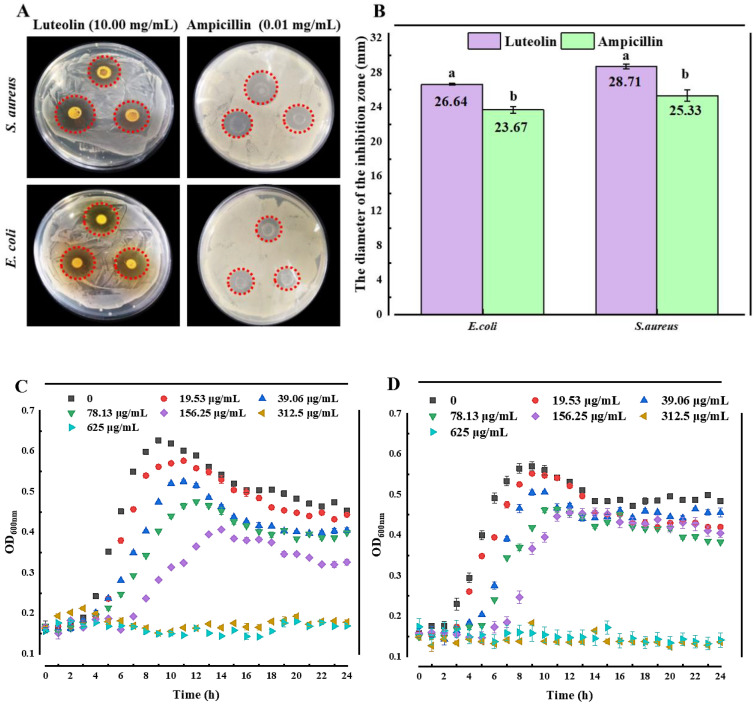
Antibacterial effects of LUT against *E. coli* and *S. aureus*. (**A**) Photos of the diameter of the inhibition zone of 10.00 mg/mL LUT and 0.01 mg/mL ampicillin for *E. coli* and *S. aureus*, respectively. (**B**) The diameter of the inhibition zone by 10.00 mg/mL LUT and 0.01 mg/mL ampicillin. Growth curves for (**C**) *E. coli* and (**D**) *S. aureus* treated with different concentrations of LUT. Different letters (a~b) mean that significant differences were observed (*p* < 0.05). The bars represent the standard deviation (*n* = 3).

The Oxford cup method was used to evaluate the antibacterial activity of LUT against *E. coli* and *S. aureus*. As shown in Figure 1A,B, LUT (10.00 mg/mL) had greater DIZ against *S. aureus* (28.71 ± 0.60 mm) and *E. coli* (26.92 ± 0.25 mm). It was significantly higher than that of ampicillin (0.01 mg/mL) against *S. aureus* (25.33 ± 0.64 mm) and *E. coli* (23.67 ± 0.38 mm) (*p* < 0.05). The results demonstrated that LUT inhibited the growth of *E. coli* and *S. aureus*. Notably, the antimicrobial activity of LUT was higher against *S. aureus* than that of *E. coli*, indicating that the differences in cell wall structures between Gram-positive and Gram-negative microorganisms could affect the antibacterial effects of this compound [15,16,17,18].

The antibacterial effects of LUT were estimated by measuring the MIC and MBC values (Table 1). As shown in Table 1, the MIC values of LUT against *E. coli* and *S. aureus* were 312.5 μg/mL, the MBC values were 625 μg/mL, and the MIC/MBC ratio was 0.5. Based on MIC and MBC results, time-dependent and concentration-dependent growth curves of *E. coli* and *S. aureus* were obtained (Figure 1C,D). The results (Figure 1C,D) showed that the inhibitory effects of different concentrations of LUT on *E. coli* and *S. aureus* were tested within 24 h. The results showed that low LUT concentrations were insufficient to inhibit the growth of *E. coli and S. aureus*. However, the growth was inhibited when treated with 625 and 312.5 μg/mL of LUT, which was consistent with the MIC and MBC results. This result suggests that LUT exhibited a high potential to inhibit these two microorganisms.

On the other hand, the OD_600_ of *S. aureus* was lower than in *E. coli* when LUT concentrations were 625 and 312.5 μg/mL, which was similar to the results of DIZ. This result indicates that the ability of LUT to inhibit *S. aureus* may be more potent than in *E. coli*. In addition, the results in Figure 1C, D also revealed the concentration and time-dependent correlation of LUT antibacterial activity against *E. coli* and *S. aureus*.

### 2.2. Antibacterial Mechanism

#### 2.2.1. Membrane Permeability Analysis

**Figure 2:**
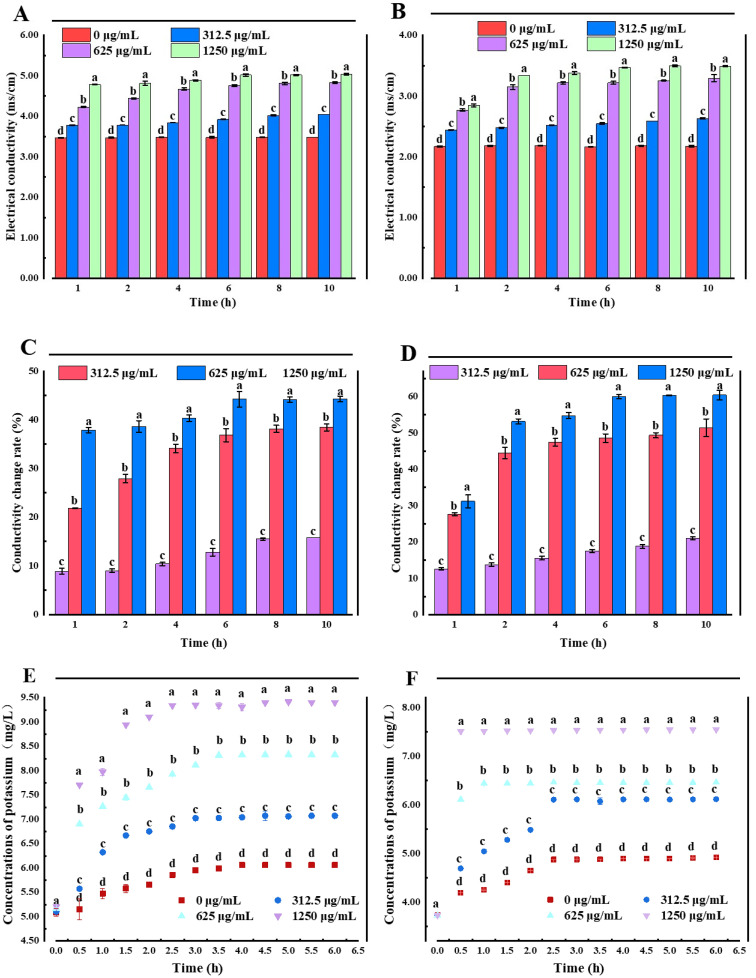
Electrical conductivity of *E. coli* (**A**) and *S. aureus* (**B**) treated with different concentrations of LUT. Changes in the conductivity rate for *E. coli* (**C**) and *S. aureus* (**D**) treated with different concentrations of LUT. The K^+^ effluxes of *E. coli* (**E**) and *S. aureus* (**F**) treated with different concentrations of LUT. Different letters (a~d) indicated significant differences between different concentrations (*p* < 0.05). Error bars indicate the standard deviation of three technical replicates (*n* = 3).

The cell membrane is a protective barrier for bacteria. When bacteriostatic agents damage this cellular structure, the protective barrier of bacteria is destroyed, and some internal electrolytes can penetrate the extracellular culture medium, increasing the conductivity of the culture medium. In addition, electrolyte extravasation obstructs various metabolic pathways in the cell, affecting the bacteria’s growth [19,20]. Therefore, the changes in the cell membrane permeability in *E. coli* and *S. aureus* after treatment with LUT were explored through the determination of conductivity. The results (Figure 2A,B) demonstrated that the electrical conductivity of bacteria after LUT treatment increased the electrical conductivity increased with the incubation time, which may be due to the fact that LUT first entered the cell wall of bacteria and attacked the cell membrane after the wall was destroyed. After the membrane was affected, the ion homeostasis was unbalanced, affecting the metabolism of the thallus, and finally leading to the death of the thallus. Moreover, the conductivity values of *E. coli* and *S. aureus* treated with 1250 μg/mL LUT were significantly higher than those treated with 625, 312.5, and 0 μg/mL LUT at the same treatment time (*p* < 0.05), indicating that higher concentrations of LUT lead to a significant cell membrane damage. Moreover, the relative conductivity of 0 μg/mL LUT-treated bacteria was unchanged during the first 4 h, followed by a slight increase, which may be due to routine bacterial cytolysis and death [21]. This trend was similar for both *E. coli* and *S. aureus*. LUT-treated *S. aureus* showed a higher rate of conductivity change than *E. coli* (Figure 2C,D), which was consistent with the results of previous antibacterial activity.

Additionally, the results in Figure 2E,F showed a K^+^ leakage in *E. coli* and *S. aureus* cells treated with different concentrations of LUT. When *E. coli* and *S. aureus* cells were treated with 312.5 µg/mL of LUT, a K^+^ efflux peak after 3 h was observed (7.02 ± 0.003 mg/L and 6.11 ± 0.000 mg/L, respectively). On the other hand, when the LUT concentration was 625 µg/mL, the maximum value of K^+^ efflux was obtained after 2.5 h (7.93 ± 0.000 mg/L and 6.46 ± 0.009 mg/L, respectively). Additionally, with the concentration of 1250 µg/mL, a stable value of K^+^ efflux was reached approximately after 1.5 h (8.95 ± 0.003 mg/L for *E. coli* and 7.52 ± 0.005 mg/L for *S. aureus*), which were significantly higher compared to the 0 µg/mL group (*p* < 0.05). This observed trend was consistent with the conductivity results. LUT caused significant and irreversible damage to the structure and membrane permeability of *E. coli* and *S. aureus*, resulting in K^+^ extravasation, which in turn inhibited cell growth [22]. Similarly, Shi and colleagues [23] showed that alkyl ferulate esters led to leakage of cell membrane components, such as K^+^, proteins, nucleotides, and β-galactosidase in *E. coli* and *Bacillus cereus*, resulting in severe damage to cell membrane permeability. These results suggested that increased membrane permeability was a determinant of the antibacterial mechanism and explain the potential importance of K^+^ release for the antibacterial activity of LUT.

#### 2.2.2. Cell Membrane Integrity

Since the disruption of cell membrane integrity can lead to cell content leakages, such as proteins and nucleic acids, the leakage of nucleic acids and proteins in *E. coli* and *S. aureus* cells after LUT treatment was determined [24]. As shown in Table 2, compared to the OD_260_ of *E. coli* and *S. aureus* in the 0 μg/mL group, the nucleic acids released by the two bacterial cells treated with different concentrations of LUT increased significantly (*p* < 0.05). In addition, the protein leakage of *E. coli* was 7.27 ± 0.25, 10.66 ± 0.69, and 20.49 ± 0.59 μg/mL after 10 h of exposure to 312.5, 625, and 1250 μg/mL of LUT, respectively. The protein permeability of *E. coli* was significantly higher than in the 0 μg/mL group (1.12 ± 0.09 μg/mL) (*p* < 0.05). Interestingly, it was observed that protein extravasation results obtained for *S. aureus* treated with LUT were similar to the results of *E. coli*. These findings suggested that LUT act on bacterial membranes, causing leakage of cellular components, such as nucleic acids and proteins, inducing bacterial death [24]. Notably, the tendency of cell content to flow out correlated with bactericidal efficacy evaluations and K^+^ results. In conclusion, the results suggest that the disruption of cell integrity may be one of the mechanisms of action of LUT.

#### 2.2.3. SEM Analysis

**Figure 3:**
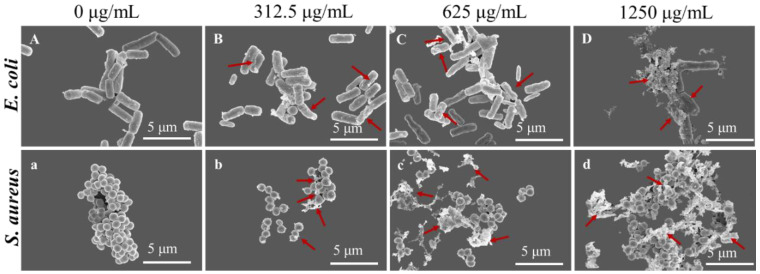
SEM images of *E. coli* and *S. aureus* treated with LUT at 0, 312.5, 625, and 1250 μg/mL for 10 h. Note: A, B, C, D: *E. coli*; a, b, c, d: *S. aureus*. Bar scale is 5 µm, magnification 24,000×. The red arrows indicate changes in the morphology of the bacteria.

The morphological changes of *E. coli* and *S. aureus* were observed by SEM. The results in Figure 3 showed that *E. coli* and *S. aureus*, without LUT treatment, exhibited typical cell structures with intact cells, smooth surfaces, rod-shaped, or spherical. However, after treatment with 312.5, 625, and 1250 μg/mL of LUT for 10 h, both bacteria showed shrinkage, depression, deformation, and lysis. Furthermore, the degree of body damage increased in a concentration-dependent manner. This result indicates that the bacterial cell membrane could be irreversibly damaged, supporting the results obtained for cellular integrity.

#### 2.2.4. CLSM Analysis

**Figure 4:**
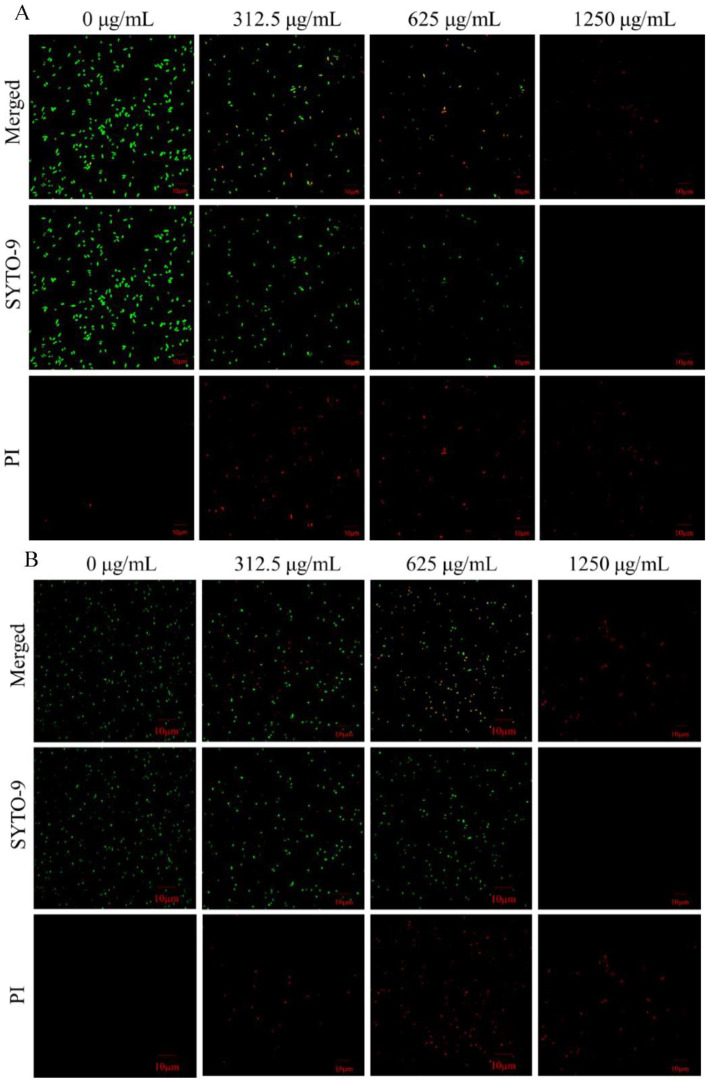
Confocal laser scanning microscopy images of *E. coli* (**A**) and *S. aureus* (**B**) treated with LUT at 0, 312.5, 625, and 1250 μg/mL for 10 h. The dead bacteria were visualized by PI staining (red fluorescence), while SYTO 9 was used to identify all bacteria (green fluorescence). When PI and SYTO 9 dye (merged) were added to the system, the insertion of PI decreases the fluorescence of SYTO 9 staining. Bacteria with intact membrane structures (green fluorescence); bacteria with damaged membrane structures (red fluorescence). The scare bar represents 10 µm.

SYTO 9 was used to label all bacteria, whether bacteria were alive or dead, and the cells appeared as green–fluorescent spots when the cell membrane was intact. PI was used to label dead cells or damage. The cells emitted a red, orange, or yellow fluorescence, depending on the degree of membrane disruption when the cell membrane was damaged [25]. After culturing *E. coli* and *S. aureus* with different concentrations of LUT for 10 h, the membrane integrity was analyzed using a live/dead cell fluorescent staining assay (Figure 4A,B). The results showed that the two bacterial cells in the 0 μg/mL group exhibited a distinct green fluorescence, indicating the presence of abundant viable bacteria. However, *E. coli* in the 0 μg/mL group exhibited a slight red fluorescence, possibly due to cell death in the normal life cycle (Figure 4A,B). On the other hand, bacteria treated with 312.5, 625, and 1250 μg/mL of LUT exhibited yellow-green or red fluorescence, and the amount was dose-dependent, indicating that the cell membrane was damaged. LUT exerted its biological activity by disrupting bacterial membrane integrity. Furthermore, the membrane disruption aggravated progressively with LUT concentration, eventually leading to cell death (Figure 4A,B). Similarly, a previous study [26] investigated the effect of protocatechuic acid on cell membrane damage in *Yersinia enterocolitica*.

#### 2.2.5. FTIR

FTIR can indirectly characterize the death induction mechanism of *E. coli* and *S. aureus* exposed to LUT, and reveal the changes in the molecular composition of the two bacteria before and after LUT treatment. The absorption peak between 3300 and 2800 cm^−1^ represented the functional group of the lipid, and the absorption peak around 1237 cm^−1^ was the phosphodiester associated with the phospholipid bilayer [12,27]. The absorption peaks of 1657, 1546, 1455 cm^−1^, and 1399 cm^−1^ represented the functional groups of the protein. Additionally, the absorption peak of 1650 cm^−1^ was amide I in α-helical structure, the absorption peak of 1546 cm^−1^ was the N-H bond of protein amide, and the absorption peak of 1455 cm^−1–^1399 cm^−1^ represented block structural protein [28]. The absorption peak of 1078 cm^−1^ represents the functional group of nucleic acid [12]. Furthermore, the absorption peak at 1235 cm^−1^ represented the asymmetric stretching of phosphodiester bonds, which was related to the phospholipid bilayer [12].

**Figure 5:**
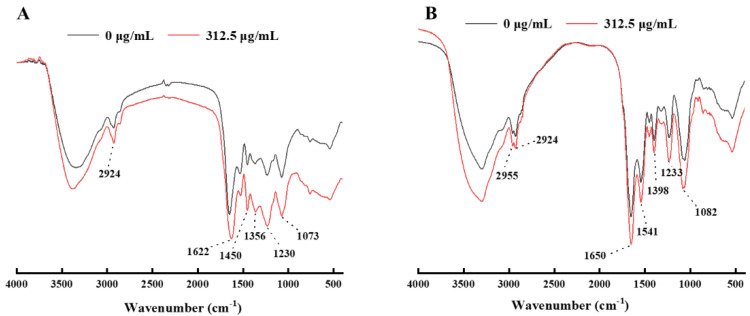
FTIR spectra of (**A**) *E. coli* and (**B**) *S. aureus* upon LUT treatments at 0, 312.5 μg/mL. The scan range was 400~4000 cm^−1^ for 32 scans.

As shown in Figure 5, the spectral characteristics of *E. coli* and *S. aureus* changed after LUT treatment. For *E. coli*, the absorption peak intensity of LUT-treated cells (at the concentration of 312.5 μg/mL at 2924 cm^−1^) increased significantly compared to the 0 μg/mL group, indicating that LUT could disrupt the phospholipid bilayer. Additionally, the intensity of the absorption peaks at 1073 cm^−1^ was enhanced, indicating that LUT can efflux nucleic acids and disrupt their structure. Additionally, the intensities of the absorption peaks at 1622, 1450 cm^−1^, and 1356 cm^−1^ increased significantly, indicating that LUT changed the secondary structure of membrane proteins.

For *S. aureus*, the peak intensities at 2955 and 2924 cm^−1^ increased after LUT treatment with 312.5 μg/mL compared to 0 μg/mL because the CH_2_ antisymmetric stretching at both places was related to the saturated lipid concentration of the membrane. Furthermore, this phenomenon supported another lipid-related band (CH_2_ bending at 1398 cm^−1^). In addition, the intensities of the absorption peaks at 1650, 1541, 1398 cm^−1^, and 1233 cm^−1^ increased, indicating that LUT changed the secondary structure of membrane proteins. Therefore, LUT inhibited the growth of bacteria by disrupting the cell membrane, which was in agreement with previous studies [29].

### 2.3. Application of LUT in Antibacterial Hand Sanitizer

**Figure 6:**
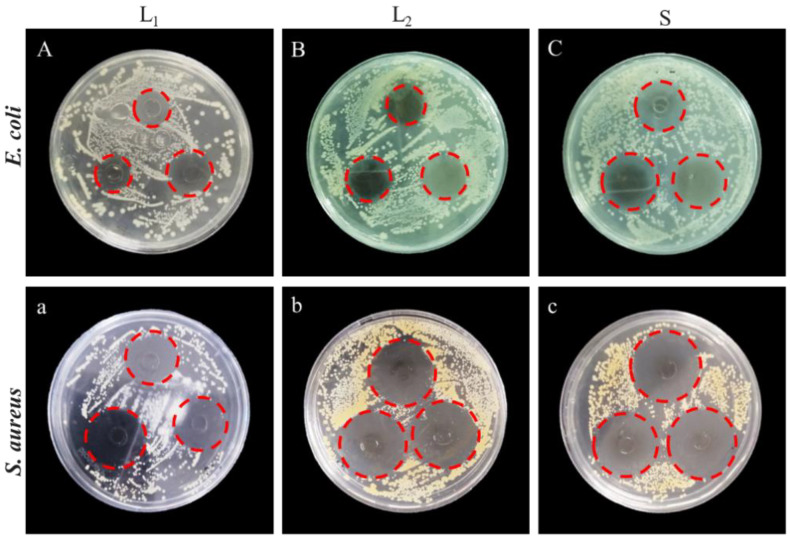
The diameter of the inhibition zone of LUT hand sanitizers compared to two commercially available hand sanitizers (L_1_ and L_2_) against *E. coli* and *S. aureus*. **A**–**C**: the diameters of inhibition zones of L_1_, L_2_ and S against *E. coli*; **a**–**c**: the diameters of inhibition zones of L_1_, L_2_ and S against *S. aureus*. Note: L_1_, the commercially available antibacterial hand sanitizer; L_2_, the commercially available common hand sanitizer.

**Figure 7:**
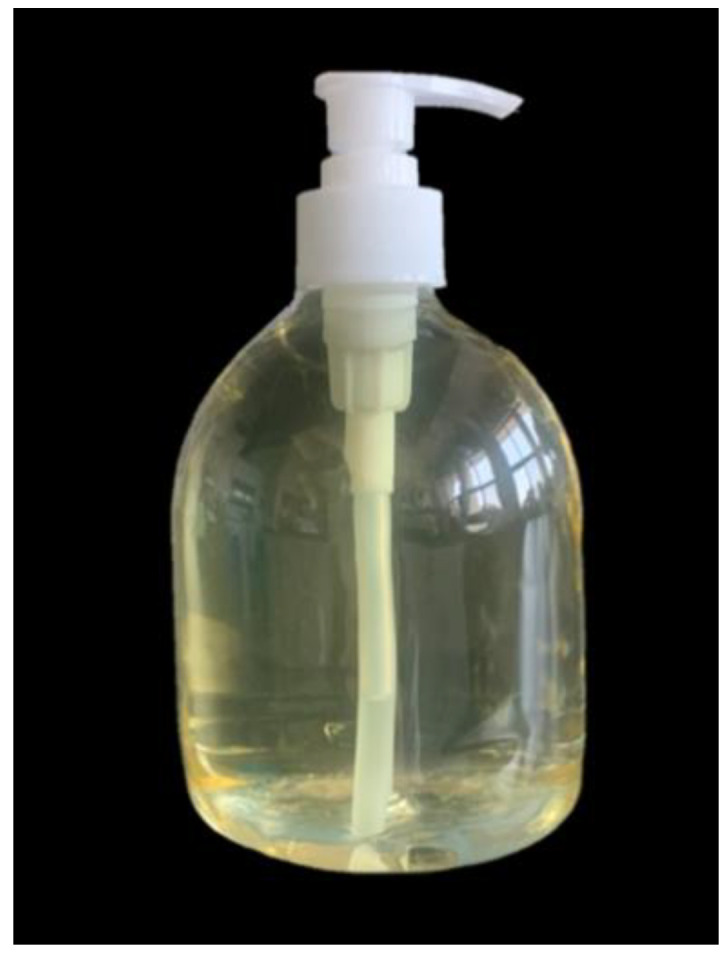
Photo of LUT antibacterial hand sanitizer product.

The antibacterial efficacy of LUT and two commercially available hand sanitizers (L_1_ and L_2_) against *E. coli* and *S. aureus* were analyzed. The results in Figure 6 showed that three hand sanitizers had inhibitory effects on both bacteria. The DIZ of LUT antibacterial hand sanitizer on *S. aureus* was 32.88 ± 0.45 mm, which was consistent with the effect of L_1_ hand sanitizer (31.64 ± 0.13 mm), significantly higher than L_2_ hand sanitizer inhibition on *S. aureus* (25.84 ± 0.39 mm, *p* < 0.05). Interestingly, the inhibition of *E. coli* by LUT hand sanitizer (29.71 ± 0.61 mm) was significantly higher than that of L_1_ and L_2_ hand sanitizers (the DIZ of L_1_ and L_2_ hand sanitizers against *E. coli* was 25.54 ± 0.12 and 22.72 ± 0.23 mm, respectively, *p* < 0.05). The DIZ of hand sanitizer against *E. coli* and *S. aureus* were listed in Table 3. As shown in Figure 7, LUT antibacterial hand sanitizer prepared in this study was yellow in appearance, clear, transparent, with no peculiar smell, and a slight walnut fragrance. In addition, pH, color, and stability were used to evaluate the quality of hand sanitizer. The pH of LUT hand sanitizer was 5.6 ± 0.18 (Table 4), which was a safe pH value for the application on the skin since it has been described that the pH should be between 4.5 and 6.5 [30]. Therefore, the skin will become scaly if the pH value is very high and alkaline. On the other hand, if the pH value is very low and acidic, the skin will be irritated [30].

In addition, the product developed in this study did not show any visible change after the heat and cold stability tests. The results showed that product brightness: L* value was 98.34 ± 0.46, which belonged to the bright degree; red and green degree: a* value was −2.57 ± 0. 59, which belonged to the green degree; yellow and blue degree: b* value was 8.24 ± 0.28, which belonged to the yellow degree.

## 3. Materials and Methods

### 3.1. Materials

LUT with ≥98% purity, extracted from walnut green husk, was purchased from Shanghai Aladdin Biochemical Technology Co., Ltd. (Shanghai, China). Ampicillin was purchased from Aladdin Reagent Co., Ltd. (Shanghai, China). Sodium polyoxyethylene ether sulfate (AES) glycerin and coconut oil diethanolamine (CAB) were bought from Shanghai Yuanye Bio-Technology Co., Ltd. (Shanghai, China). All other reagents were of an analytical grade and purchased from Sinopharm Chemical Reagent Co., Ltd. (Yangling, China).

### 3.2. Strains and Culture Conditions

*E. coli* (ATCC 25922) and *S. aureus* (ATCC 29213) were supplied by the College of Food Science and Engineering, Northwest A&F University (Yangling, China). *E. coli* and *S. aureus* were activated in LB medium and were incubated under aerobic conditions at 37 °C for 24 h. *E. coli* and *S. aureus* were cultured in LB broth and incubated at 37 °C for 18–20 h. Next, the bacterial suspensions obtained by centrifugation (6000× *g*, 10 min, 4 °C) were washed three times with pH 7.3 phosphate buffer solution (PBS) and resuspended in LB broth to 10^8^ CFU/mL (OD_600nm_ = 0.5) for further investigation.

### 3.3. Determination of the Diameter of the Inhibition Zone (DIZ)

The antibacterial effects of LUT against *S. aureus* and *E. coli* using the Oxford cup method were analyzed [31]. The suspensions containing ~10^8^ CFU/mL bacteria were prepared and cultured on the surface of the LB plate. After the agar solidified, 200 μL of the LUT solution (10.00 mg/mL) and ampicillin (0.01 mg/mL) were filled with the Oxford cup (a stainless cylinder, outer, inner diameter, and height were 8.0, 6.0, and 10.0 mm, respectively). After incubation at 37 °C for 24 h, the DIZ around samples were conducted in triplicate by the vernier caliper (accuracy of 0.02 mm).

### 3.4. Determination of Minimum Inhibitory Concentration (MIC) and Minimal Bactericidal Concentration (MBC)

The MIC and MBC were analyzed by the two-fold dilution method [32]. Briefly, 8 test tubes were numbered 1–8, 10 mL LB broth medium was added to the first test tube, and 5 mL of broth medium was added to the remaining test tubes. Then, 100 μL LUT solution was added to the no. 1 test tube, and a gradient dilution was performed. After, the LUT concentrations in the 8 test tubes were measured and ranged from 10 to 2000 μg/mL. Finally, 65 μL of bacterial suspension (10^8^ CFU/mL) was added to each test tube. This study used LB broth medium as the blank control, and 2% ethanol (*v*/*v*) without LUT was used as a negative control. After 24 h in the culture at 37 °C, a turbid medium would be considered to have bacterial growth, and the critical tube concentration for sterile growth was the MIC. Finally, 0.2 mL of bacterial liquid was taken from each sterile growth tube, coated in a solid medium, and incubated at 37 °C for 24 h. The concentration of the sterile growth tube on the plate was MBC.

### 3.5. Determination of Growth Curves

The growth curves were constructed by the method described by Shi and colleagues [33]. Briefly, 150 μL of *E. coli* and *S. aureus* (10^8^ CFU/mL) were added to a 100-well honeycomb plate, respectively. According to the determined MIC concentration, 150 μL of LUT solution was added, so that the concentration of LUT solution in the final medium was 0, 19.53, 39.06, 78.13, 156.25, 312.5, and 625 μg/mL, and the ethanol content was the same. Optical density measurements at 600 nm (OD_600_) measurements were taken for 1 h in the automated Bioscreen C system (Labsystems, Helsinki, Finland). An equal amount of absolute ethanol was added as the control group; the experiment was repeated 3 times, and the average value was taken. Taking the time (t) as the abscissa and the OD value as the ordinate, the growth curves of *E. coli* and *S. aureus* treated with LUT were plotted.

### 3.6. Extracellular Conductivity

The ion permeability of the bacterial cell membrane was analyzed by the extracellular conductivity [34]. *E. coli* and *S. aureus* (10^8^ CFU/mL) were added to 200 mL of fresh LB broth liquid medium at 2% inoculum and incubated for 16 h. LUT solution was added to make the final concentrations 0, 312.5, 625, and 1250 μg/mL, respectively. The cultures were incubated for 1 h, 2 h, 4 h, 6 h, and 8 h; 5 mL of the culture medium was centrifuged at 6000× *g* for 10 min, and the conductivity of the supernatant was measured with a conductivity meter (DDS-307A, Shanghai, China) conductance meter. The experiment was repeated three times and the average value was taken.

### 3.7. Extracellular Potassium Ion Concentration

The leakage of intracellular potassium (K^+^) to the supernatant was determined according to a previous methodology [35]. Briefly, *E. coli* and *S. aureus* cells were washed and resuspended in PBS solution. After, the suspension was incubated with different LUT concentrations for 10 h. After, the mixture was filtered using a 0.22 μm PVDF membrane to remove bacterial cells. Finally, an atomic absorption spectrophotometer (ZEEnit 700P, Jena, Germany) determined the concentration of potassium ions in the supernatant. In this study, different concentrations of KCl solutions were used as standard curves.

### 3.8. Cell Membrane Integrity

The membrane integrity of bacterial cells was assessed by analyzing the release of bacterial cell components, such as nucleic acids and proteins, in cell suspensions [36]. Therefore, the logarithmic growth phases of *E. coli* and *S. aureus* were treated with LUT at 312.5, 625, and 1250 μg/mL and incubated at 37 °C. After 8 h incubation, samples were collected. Then, samples were centrifuged at 6000× *g* for 10 min at 4 °C, and the supernatant was used to detect the amount of nucleic acid and protein released. The amount of nucleic acid was defined as the absorbance value at 260 nm (OD_260nm_). The protein concentration was obtained using the Bradford protein assay kit (Solarbio, Beijing, China). Furthermore, 0 μg/mL of sample was used as the control, and the results were expressed as μg/mg.

### 3.9. Scanning Electron Microscopy (SEM)

In this study, SEM was performed to observe morphological changes in *E. coli* and *S. aureus* at 0, 312.5, 625, and 1250 μg/mL [37]. Briefly, the treated cell suspension was fixed with 2.5% glutaraldehyde for 10 h and dehydrated with different concentrations of ethanol (10, 30, 50, 70, 80, 90, and 100%) for 10 min, followed by drying at the critical point for more than 4 h and spray treatment. Finally, the samples were analyzed by SEM (Nano SEM-450; FEI, Hillsboro, OR, USA).

### 3.10. Confocal Laser Scanning Microscopy (CLSM)

For this experiment, 5 mL of bacterial suspension (approximately 10^8^ CFU/mL) was centrifuged at 6000× *g* for 10 min, as previously described [38]. Then, the supernatant was discarded, and the precipitated cells were resuspended in a sterile PBS solution (pH 7.3). After, cells were treated with different LUT concentrations, 0, 312.5, 625, and 1250 μg/mL, for 8 h at 37 °C. After the cells were washed with sterile PBS solution (pH 7.3), and centrifuged at 6000× *g* for 10 min, these steps were repeated three times to obtain bacterial cells. Then, 200 μL of propidium iodide (PI) and SYTO 9 working dye solutions were added to the bacterial suspension and incubated in the dark for 15 min. Finally, the bacterial droplets were placed on a glass slide, covered with a coverslip, and the droplets were placed in the CLSM (FV1200; Olympus, Tokyo, Japan), and the 1000× magnification was used.

### 3.11. Fourier Transform Infrared Spectroscopy (FTIR)

The changes in cell membrane components were measured by FTIR. First, processed samples were collected. Then, the cell precipitate was resuspended in 1 mL of PBS solution (pH 7.3) and placed at −80 °C for 12 h. Then, a freeze dryer was used for 48 h. In this experiment, the tablet method was used to test. 100 mg of dried potassium bromide was mixed with 1.0 mg of the dried sample to be tested, fully ground, and then the mixed powder was pressed into a circular sheet with a thickness of approximately 1 mm. Finally, the spectra of the samples were obtained by the Bruker vertex 70 FTIR (Bruker, Karlsruhe, Germany). The scan range was 400~4000 cm^−1^ for 32 scans [15].

### 3.12. Product

#### 3.12.1. Formulations of Hand Sanitizers

LUT antibacterial hand sanitizers (denoted as S) were prepared as the following formula (*w*/*v*): AES (10.0%, *v*/*v*) and mixed with CAB (2.0%, *v*/*v*) in a 250 mL beaker. Then, 60 mL of deionized water was added, heated at 80 °C, stirred until completely dissolved, and cooled to 40 °C. Then, NaCl (2%, *v*/*v*), citric acid (0.6%, *v*/*v*), glycerol (9.0%, *v*/*v*), LUT solution (312.5 μg/mL, 8.0%, *v*/*v*), flavor (0.001% *v*/*v*) were added to a 250 mL beaker. Finally, water was used to complete the final.

The main antibacterial ingredient of the antibacterial effects of the commercially available antibacterial hand sanitizer (denoted as L_1_) is salicylic acid, and other ingredients are water, sodium coconut polyether carbonate, sodium sulfate, sodium chloride, disodium EDTA, and so on. The main ingredients of the commercially available common hand sanitizer (denoted as L_2_) are water, sodium coconut polyether sulfate, lauramidopropyl betaine, sodium sulfate, PEG-7 glyceryl cocoate, sodium benzoate, diphenylketone-4methylchloroisothiazolinone, disodium EDTA, magnesium nitrate, CI42090, CI6185, and so on.

#### 3.12.2. Antibacterial Effect Comparison

The DIZ of L_1_, L_2_, and S were determined by the Oxford cup method according to Section 3.3.

#### 3.12.3. Stability Evaluation

The prepared LUT hand sanitizer was diluted with boiled distilled water and cooled to 25 °C. A sample solution of 1:10 (mass concentration) was prepared and mixed to be tested. The pH of the sample was measured using a pH meter and these measurements were performed 3 times. The difference between measurements was less than 0.1 pH unit. The results were expressed as the average of three measurements, with an accuracy of 0.1.

In this experiment, the cold resistance stability was determined by placing the hand sanitizer in a refrigerator at −5 ± 2 °C. After 24 h, the samples were removed and placed at room temperature (25 °C) to observe the morphology of the samples.

Additionally, to evaluate heat resistance stability, the hand sanitizer was placed in a constant temperature incubator of 40 ± 1 °C. After 24 h, the product was removed and placed at room temperature (25 °C) to evaluate significant differences in product morphology.

In this study, the color difference was evaluated, and for that, 3 mL of the sample was transferred to a centrifuge tube and centrifuged at 1000× *g* for 5 min to remove air bubbles in the product. Then, the sample was placed in a cuvette, and a colorimeter photometer (Shanghai, China) was used. This equipment was used in a transmission mode. After the sample was placed in the transmission light port, covered with a photomask, and the lightness value (L*, 0–100), a* value (-a* greenness, +a* redness), and b* value (-b* blueness, +b* yellowness) were recorded. The samples were remixed after each measurement, and the procedure was repeated 3 times.

### 3.13. Statistical Analysis

All experiments were conducted in triplicate, and the results were presented as mean ± standard deviation (SD). Statistical analysis was performed by IBM SPSS Statistics 20.0 software (SPSS, Inc., Chicago, IL, USA). Means were evaluated using Duncan’s test, and a *p* < 0.05 was considered statistically significant. Origin version 2019 was used for graphs (Northampton, MA, USA).

## 4. Conclusions

These results showed that LUT had antibacterial activities against *E. coli* and *S. aureus*. The antibacterial activities increased with the increase of LUT concentrations with MIC 312.5 μg/mL. Furthermore, LUT increased membrane permeability, induced leakage of cellular contents, and disrupted cell membrane integrity. In addition, the results also revealed that the application of LUT in hand sanitizer markets could be promising. However, more studies are needed to fully explore the value of the LUT hand sanitizer to know its applicability and limitations in the future.

## Figures and Tables

**Table 1:** MIC, MBC, and MBC/MIC ratio of LUT against *E. coli* and *S. aureus*.

Organism	LUT Concentration (μg/mL)		
	MIC Value	MBC Value	MIC/MBC Ratio
*S. aureus*	312.5	625	0.5
*E. coli*	312.5	625	0.5

Note: MIC, minimum inhibitory concentration; MBC, minimal bactericidal concentration.

**Table 2:** Effects of LUT on extracellular protein and nucleic acids of *E. coli* and *S. aureus*.

Concentration (μg/mL)	Nucleic Acids (OD_260 nm_)		Protein (μg/mL)	
	*E. coli*	*S. aureus*	*E. coli*	*S. aureus*
0	0.26 ± 0.004 ^d^	0.22 ± 0.003 ^d^	1.12 ± 0.09 ^d^	1.46 ± 0.05 ^d^
312.5	0.28 ± 0.002 ^c^	0.27 ± 0.004 ^c^	7.27 ± 0.25 ^c^	9.05 ± 0.10 ^c^
625	0.31 ± 0.002 ^b^	0.29 ± 0.007 ^b^	10.66 ± 0.69 ^b^	11.15 ± 0.29 ^b^
1250	0.34 ± 0.001 ^a^	0.33 ± 0.006 ^a^	20.49 ± 0.59 ^a^	21.68 ± 0.20 ^a^

Note: The different letters (a~d) in each column represent significant differences (*p* < 0.05).

**Table 3:** Diameter of inhibition zone of L_1_, L_2_, and S hand sanitizer against *E. coli* and *S. aureus*.

Group	*S. aureus* (mm)	*E. coli* (mm)
L_1_	31.64 ± 0.13 ^a^	25.54 ± 0.12 ^b^
L_2_	25.84 ± 0.39 ^b^	22.72 ± 0.23 ^c^
S	32.88 ± 0.45 ^a^	29.71 ± 0.61 ^a^

Note: L_1_-the commercially available antibacterial hand sanitizer; L_2_- the commercially available common hand sanitizer; S-LUT antibacterial hand sanitizer. The different letters (a~c) in each column represent significant differences (*p* < 0.05).

**Table 4:** Stability evaluation of LUT antibacterial hand sanitizer.

Analysis	LUT Antibacterial Hand Sanitizer		
pH value	5.6 ± 0.18		
Cold resistance stability	Not solidified		
Heat resistance stability	Not discolored		
Color	L*: 98.34 ± 0.46	a*: −2.57 ± 0. 59	b*: 8.24 ± 0.28

Note: the lightness value (L*, 0–100), a* value (-a* greenness, +a* redness), and b* value (-b* blueness, +b* yellowness).

## Data Availability

Data is contained within the article and is available at request from the corresponding author.
